# Advancing Treatment in Pediatric Multiple Sclerosis: The Promise of B-Cell-Targeting Therapies

**DOI:** 10.3390/ijms26135989

**Published:** 2025-06-22

**Authors:** Charalampos Skarlis, Maria Kotsari, Maria Anagnostouli

**Affiliations:** 1 Research Immunogenetics Laboratory, First Department of Neurology, School of Medicine, National and Kapodistrian University of Athens, Aeginition University Hospital, Vas. Sofias 72-74, 11528 Athens, Greece; charskarlis@med.uoa.gr (C.S.);; 2 Multiple Sclerosis and Demyelinating Diseases Unit, Center of Expertise for Rare Demyelinating and Autoimmune Diseases of CNS, First Department of Neurology, School of Medicine, National and Kapodistrian University of Athens, Aeginition University Hospital, 11528 Athens, Greece

**Keywords:** pediatric-onset multiple sclerosis, rare demyelinating CNS diseases, B-cells, Rituximab, Ofatumumb, Alemtuzumab, Ocrelizumab, BTKis

## Abstract

Pediatric-onset multiple sclerosis (POMS) is a rare yet increasingly recognized demyelinating disease of the central nervous system, characterized by a highly inflammatory disease course and an elevated relapse rate compared to adult-onset MS (AOMS). Given the unique immunopathogenesis of POMS, recent therapeutic strategies have shifted toward early initiation of high-efficacy disease-modifying therapies (DMTs) to minimize irreversible neurological damage. Among these, B-cell-targeting therapies, particularly anti-CD20 monoclonal antibodies, have shown efficacy in adult MS and are emerging as promising candidates for POMS treatment. The present review summarizes the current knowledge of the role of B-cells in POMS pathophysiology and evaluates the therapeutic potential of anti-CD-20 agents. It also highlights ongoing clinical trials and future perspectives, including novel B-cell-directed approaches such as anti-CD19 therapies, Bruton’s tyrosine kinase (BTK) inhibitors, and BAFF-targeting agents.

## 1. Introduction

Early-onset-multiple sclerosis, including pediatric- and adolescent-onset multiple sclerosis (MS), accounts for approximately 3–5% of all MS cases, with the term pediatric-onset MS (POMS) largely being used mutually for both age groups. POMS is considered a rare demyelinating disease of the central nervous system (CNS) [1,2]. However, its burden is increasing worldwide, with an annual incidence ranging from 0.05 to 2.85 per 100,000 patients and a prevalence varying between 0.69 and 26.92 per 100,000 children <16 or 18 years old [3,4], while the hypothesis of a genetic predisposition for MS is supported by the 25.4% risk of disease development in monozygotic twins [5]. The mean age of disease onset in pediatric patients is mostly between 13 and 16 [3]. Notably, its prevalence is lower in prepubescent patients (i.e., <12 years old) [6,7]. Until 12 years of age, POMS prevalence is balanced between sexes. However, the female-to-male ratio increases after puberty, possibly due to the influence of sex hormones on the immune system [8,9].

Similarly to AOMS, several genetic, epigenetic, and hormonal risk factors have been linked to POMS development [10,11]. Of note, genetic linkage studies performed mostly in AOMS have revealed a strong association between the human leukocyte antigen (HLA) region and MS risk. In particular, the HLA-DRB*15:01 allele represents the strongest genetic risk factor for the development of both AOMS and POMS subjects compared to the general population [10,12,13,14,15]. Of importance, a recent study by our research group revealed that the *E2* allele of the apolipoprotein E (ApoE) gene is associated with higher MS risk in pediatric patients compared to both adult patients and controls [16], suggesting that besides the mostly common genetic backgrounds, AOMS and POMS, distinct gene variants may drive the clinical differences between these two MS entities.

Moreover, vitamin D deficiency, obesity, and smoking—both active and passive—have been associated with an increased risk for MS [17,18,19,20].

Finally, infectious agents such as the Epstein–Barr Virus (EBV) have been strongly associated with increased susceptibility for POMS independently of age, sex, race, ethnicity, and HLA-DRB1 status [19].

Despite the similarities, the two MS entities display several different features that rule the therapeutic strategy for these patients (Table 1). Compared to AOMS patients, POMS patients display a more inflammatory and aggressive disease onset, showing a high number of demyelinating lesions and disease activity, along with a higher relapse rate (2.3–2.8 times higher), but they also show slower long-term disability accumulation, although, ultimately, they are usually at a younger age. Axonal damage is more pronounced in pediatric patients, indicating a more severe disability course compared to adults. Additionally, POMS patients almost exclusively display the relapsing–remitting disease form and accumulate disability at a slower rate compared to AOMS patients. However, POMS patients shift to a secondary progressive disease course approximately ten years earlier than AOMS patients [21,22,23,24].

In this context, several therapeutic agents aim to delay disease progression and disability accumulation for as long as possible. Great research efforts have revealed the pivotal role of B-cells in MS pathogenesis, and several B-cell-targeting agents have emerged as promising therapies for both AOMS and POMS.

The present narrative review aims to summarize the current knowledge on the role of B-cells in POMS pathophysiology and evaluates the therapeutic potential of B-targeting therapies.

## 2. The Role of B-Cells in Multiple Sclerosis

The traditional understanding of MS pathophysiology suggests that aberrantly activated and/or dysregulated pro-inflammatory autoreactive CD4^+^ and CD8^+^ T-cells are the main mediators of CNS inflammation, which ultimately results in perivascular demyelination, glial cell activation, and neuroaxonal injury [25,26].

**Figure 1:**
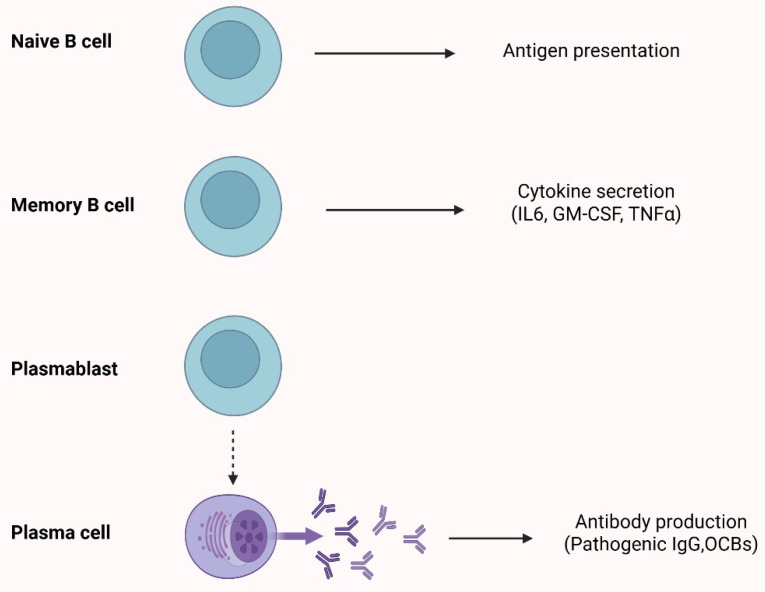
Schematic overview of the role of B-cells in multiple sclerosis. TNFα: tumor necrosis factor alpha; GM-CSF: granulocyte-macrophage colony-stimulating factor; OCBs: oligoclonal bands.

Of interest, over the past decade, mounting evidence supports the major role of B-cells in both inflammatory and neurodegenerative aspects of the disease [27,28]. In this context, increased immunoglobulin (Ig) synthesis rates, cerebrospinal fluid (CSF) oligoclonal bands (OCBs), and antibodies bound to myelin fragments within phagocytic cells in the CNS parenchyma are some of the earliest indications of the B-cell involvement in MS (Figure 1) [29,30,31].

The contribution of B-cells in disease pathophysiology is also supported by recent genetic studies. In addition, the HLA-DRB1*03 allele—previously linked to humoral immunity activation in the context of Neuromyelitis Optica—has been found in significantly higher frequency in POMS patients compared to both AOMS and the general population [14]. Additionally, the rs1041569 TT B-cell activating factor (BAFF) gene variant [32] and the H159Y polymorphism rs61756766, encoding the BAFF receptor [33], have been linked with increased MS risk.

Moreover, the key role of B-cells in antigen presentation and pro-inflammatory cytokine production—including IL-6, granulocyte-macrophage colony-stimulating factor (GM-CSF), and tumor necrosis factor-α (TNF-α) [25,26,27]—and the detection of CD20^+^ B-cells in the perivascular space of brain vessels [28] have made B-cells a promising therapeutic target.

As a result, several therapeutic agents targeting cluster of differentiation (CD) molecules expressed on the surface of B-cells during the different stages of their maturation have been proposed as promising therapeutic targets [34,35,36].

B-cell maturation is an antigen-independent process starting from the hematopoietic stem cells in the bone marrow and followed by antigen-dependent maturation in the peripheral lymphoid tissues [30,31,37]. Pre-B-cells (CD19^+^ and CD20^+^), originating from pro-B-cells (CD19^−^ and CD20^−^), develop into immature B-cells in the bone marrow, at which point IgM is expressed. These cells then evolve into mature B-cells following activation by their cognate antigen and co-stimulatory factors. Ig isotype switching after activation occurs in germinal centers, which stimulates B-cells to migrate to various locations (including the bone marrow, brain, gut, spleen, and tonsils), where they differentiate into either memory B-cells (CD27^−low^) or plasmablasts (both early/CD27^−high^ and CD40L^+^ plasmablasts and late/CD27^+^ and CD38^+^ plasmablasts) [31]. Finally, under the effect of specific chemokines, including CXCL12, CCL25, and CCL28, they differentiate in antibody-producing plasma cells. In these locations, B-cells also act as antigen-presenting cells or produce pro-inflammatory cytokines, which enhance inflammatory processes [38].

Activated B-cells normally interact with T helper (Th) cells in germinal centers, where they differentiate into memory B-cells and induce Th effector activation. It is hypothesized that, in MS, peripheral B-cells escape the control of regulatory T-cells, which are functionally impaired [31]. Highly pathogenic B- and T-cells can migrate through the blood–brain barrier, expressing distinct chemokine receptors, pro-inflammatory cytokines, and adhesion molecules, where they become reactivated and cause MS-related CNS pathology [31]. However, the precise mechanism by which these cells contribute to the MS-related abnormal immune response remains incompletely understood.

Of note, compared to AOMS, pediatric patients may exhibit differences in peripheral B-cell maturation, trafficking, and function due to age-dependent immune system development and hormonal modulation during puberty [8,9,39]. For instance, the heightened pro-inflammatory response in POMS could reflect an exaggerated B-cell cytokine profile or reduced regulatory B-cell activity, both of which merit further investigation. Additionally, sex hormone fluctuations may contribute to altered B-cell survival and antigen presentation in adolescence [40]. Importantly, the long-term immunological consequences of depleting B-cells during immune system maturation are not fully elucidated. Future studies should aim to characterize pediatric-specific B-cell subsets, CNS infiltration profiles, and their longitudinal response to targeted therapies to guide safer and more effective treatment strategies in this unique population.

## 3. Methodology

The present narrative review was conducted through a targeted literature search using the PubMed, Scopus, Cochrane, and ClinicalTrials.gov databases, including only English-language articles published from January 2013 to April 2025. Keywords included the following: “pediatric multiple sclerosis”, “POMS”, “B-cells”, “CD20”, “rituximab”, “ocrelizumab”, “ofatumumab”, “BTK inhibitors”, “BAFF”, “inebilizumab” and “anti-CD19”. Published articles were selected based on clinical relevance, with a focus on B-cell-directed therapies and inclusion of pediatric or adolescent MS patients.

## 4. Therapeutic Agents Targeting B-Cells and Their Use in the Treatment of POMS

**Figure 2:**
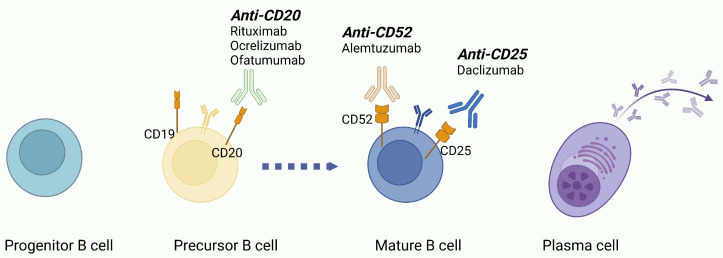
B-cell ontology and B-cell-targeting therapies for pediatric-onset and adult-onset multiple sclerosis treatment.

B-cell-targeting therapies have revolutionized the MS treatment approach (Figure 2) [41]. B-cell-depleting therapeutic strategies have primarily focused on the development of CD20-targeting monoclonal antibodies (e.g., Rituximab, Ocrelizumab, and Ofatumumab), which effectively deplete B-cells and reduce disease activity [30,36]. Moreover, novel agents, including anti-CD19 (e.g., Inebilizumab) and CD22/CD52-directed therapies, target broader B-cell subpopulations (Table 2). Other emerging agents, such as Bruton’s tyrosine kinase (BTK) inhibitors, further expand precision immunotherapy, offering promising alternatives for both AOMS and POMS management [42,43].

### 4.1. Anti-CD20 Agents: Mechanism of Action

CD20 is a transmembrane tetramer molecule present on the surface of particular subsets of B-cells, ranging from pre-B-cells to early plasmablasts [34,44,45]. CD20 seems to play a role as a cytoplasmic calcium downstream regulator of the interaction between the B-cell receptor (BCR) and antigens [44].

Anti-CD20 monoclonal antibodies (mAbs) bind to the CD20 molecule, causing B-cell depletion [34]. According to their mechanisms of action, they are classified into two groups, type 1 and type 2. Type 1 refers to anti-CD20 mAbs eliminating B-cells potentially via hyper cross-linking to CD20, causing B-cell classical apoptosis through the caspase-dependent pathway, promoting both complement-dependent cellular cytotoxicity (CDCC) and antibody-dependent cellular cytotoxicity (ADCC) [44,46]. Contrariwise, type 2 anti-CD20 mAbs induce B-cell depletion only through induction of apoptosis and ADCC.

Rituximab and Ocrelizumab are both classified as type 1 mAbs and have overlapping binding sites in the large loop of the CD20 molecule. Unlike Rituximab, Ocrelizumab is more active in the ADCC pathway than CDCC [44,46,47]. The main clinical trials regarding B-cell-targeting therapeutics in POMS are summarized in Table 3.

### 4.2. Rituximab

Rituximab is a monoclonal chimeric antibody targeting CD20 that was initially used for the treatment of B-cell lymphoma [48,49] and, thereafter, for the treatment of rheumatoid arthritis (RA) [50] and systemic vasculitis [51]; subsequently, it has been used off-label in several other autoimmune disorders, including the CNS demyelinating diseases [52]. Over the last fifteen years, several case reports, case series, and cohort studies on pediatric patients with inflammatory CNS diseases (including MS), treated with Rituximab, have been published [53,54,55]. However, data about the efficacy and safety of Rituximab only in POMS patients are very limited.

Salzer et al. retrospectively described a case series of 14 POMS patients treated with Rituximab for 1–48 months (mean duration of therapy: 23.6 months). Rituximab treatment showed efficacy, achieving a reduction in, or stabilization of, EDSS score (6/14 patients) and clinical relapses, as well as facilitating the absence of MRI activity in 13/14 patients, with only 1 patient developing new MRI lesions after six months of therapy. However, Rituximab was discontinued for two individuals due to skin reactions and new non-enhancing MRI lesions [56,57].

Recently Breu and colleagues retrospectively described a cohort of 61 POMS patients treated with rituximab for 61 POMS patients. The majority of POMS patients (42/61) had been treated with Rituximab as a first-line therapy. Rituximab showed efficacy in achieving NEDA-3 (no evidence of disease activity-3 defined as the absence of relapses, absence of MRI activity, and absence of EDSS progression) in 70% (35/50) of patients during the treatment period. Moreover, 93% of patients remained free from relapses, 77% remained free from new T2 lesions, and 100% were free from contrast-enhanced lesions [58].

Regarding the safety of treatment, fever, infusion reactions, headache, rash with morbilliform exanthema, and pruritus are the most common side effects associated with Rituximab. Moreover, progressive multifocal leukoencephalopathy—the most severe complication of therapies targeting B-cells—has also been reported [57].

In addition, McAtee et al. recently carried out a study on a cohort of 468 patients aged <21 years receiving Rituximab for several conditions, mainly reporting mild adverse events (such as infusion reactions and infections). Of note, prolonged neutropenia and PML were not observed in this cohort, suggesting that these potentially life-threatening conditions are rare in the pediatric population [59].

### 4.3. Ocrelizumab

Ocrelizumab is a recombinant humanized anti-CD20 monoclonal antibody approved for both relapsing (RRMS) and primary progressive MS (PPMS) (the first ever approved therapy for PPMS), reducing inflammatory disease activity and slowing disability and MRI progression, as shown in phase III randomized controlled clinical trials [60,61,62].

Regarding POMS patients, the results of the OPERETTA I (NCT04075266) study—an open-label, parallel group, phase II trial investigating the pharmacokinetic and pharmacodynamic properties, safety, and tolerability of Ocrelizumab in children and adolescents with relapsing–remitting MS (RRMS) aged 10–17 years—were published very recently. The study design consisted of four phases: screening (up to 8 weeks), a dose exploration period of 24 weeks, an optional OCR extension period (OOE; 264 weeks), and safety follow-up of a minimum of 48 weeks. Patients with a body weight (BW) ≥ 25 kg and <40 kg were assigned to cohort 1 to receive 300 mg Ocrelizumab IV every 24 weeks; patients with a BW ≥ 40 kg were assigned to cohort 2 to receive 600 mg Ocrelizumab IV every 24 weeks [63].

Ocrelizumab treatment with the dose of 600 mg led to rapid CD19^+^ B-cell depletion, which was sustained throughout the study and was similar to the data collected from the OPERA I/II trials in adult RRMS patients who received the same dose. Ocrelizumab showed efficacy in the control of disease activity, and no relapses occurred. Moreover, EDSS score reduction was registered from baseline to Weeks 48 and 96 in both treatment groups, and NEDA was observed in the majority of patients at Weeks 48 and 96 [62].

Currently, there is an ongoing phase III randomized, double-blind, double-dummy, multicenter study (WN42086, NCT05123703) evaluating the safety and efficacy of Ocrelizumab administered by intravenous (IV) infusion every 24 weeks compared to Fingolimod (taken orally daily) in children and adolescents with RRMS aged between 10 and 18 years. As a primary outcome, this study aims to evaluate the treatment effect on Annualized Relapse Rate (ARR) from the baseline up to approximately 4 years [64].

### 4.4. Ofatumumab

Ofatumumab is a fully human IgG1 monoclonal antibody that targets a distinct small loop epitope on the CD20 molecule, which is a different epitope from Rituximab’s target, leading to the depletion of circulating CD20 B-cells. Acting via both ADCC [65] and CDCC [66], Ofatumumab is considered a more potent activator of complement-dependent cytotoxicity in vitro [67].

The ASCLEPIOS I (NCT02792218) and II (NCT02792231) trials assessed the efficacy of Ofatumumab versus Teriflunomide in adult patients with relapsing forms of MS displaying a significantly lower AAR compared to MS patients treated with Ofatumumab [68]. Ofatumumab achieved a significant decrease in gadolinium-enhancing MRI lesions, as well as significant disability reduction [69].

The benefit–risk profile of Ofatumumab compared to Teriflunomide has been assessed in a subpopulation of recently diagnosed and treatment-naïve (RDTN) MS patients derived from the combined cohort of the ASCLEPIOS I and II trials [70]. This analysis showed that Ofatumumab achieved an ARR reduction of 50%, delayed the confirmed disability worsening (CDW) at 6 months by 46%, and improved 6-month progression independent of relapse activity (PIRA) by 56% compared to the Teriflunomide-treated group. Additionally, the adverse events were mild (nasopharyngitis, injection-related systemic reactions, headache, and upper respiratory tract infections) in both groups and similar to those recorded in the ASCLEPIOS I and II trials.

The long-term safety of Ofatumumab was assessed in the ALITHIOS (NCT03650114) study, which included patients who completed the ASCLEPIOS I and II trials [71]. Adverse events were reported in 83.8% of the participants, and 9.7% of the patients treated with Ofatumumab. PML or other opportunistic infections were not identified, and the risk of malignancy was low (0.6%). The majority of the adverse events reported were injection site-related. Consequently, Ofatumumab has been approved by the FDA for the treatment of adult RRMS and SPMS patients, while the EMA has approved Ofatumumab only for RRMS [66].

However, in the pediatric MS population, only limited real-world data derived from case series are available. Very recently, Kuzminykh and colleagues described three POMS patients who switched from first-line disease modifying therapies (DMTs) to Ofatumumab [72]. Ofatumumab showed short-term efficacy in all treated patients, reducing clinical and radiological activity. Moreover, mild and manageable adverse events, such as hyperthermia and headache, were observed after the first injection, which these events being resolved using antipyretic and antihistamine medication.

Currently is active (not recruiting yet) a phase II, three-arm, randomized, double-blind, active-controlled (Fingolimod) trial (NCT04926818, NEOS) aiming to evaluate the efficacy and safety of Ofatumumab and Siponimod compared to Fingolimod. The primary outcome is the asses of ARR changes from the baseline up to 24 months [73].

## 5. Anti-CD25 Agents: Daclizumab

Daclizumab is an IgG1 monoclonal antibody (MAb) targeting the interleukin (IL)-2 receptor alpha (IL-2Rα) chain (CD25), thus blocking the binding of IL-2 to its receptor, leading to a reduction in activated T-cells [74]. Daclizumab’s unique mechanism of action raised great expectations; however, it was retracted from the market after its approval for adult-onset RRMS due to its association with numerous events of fatal encephalitis [75]. Up to today, no new clinical trials re-evaluating the safety of Daclizumab are active.

Regarding the pediatric MS population, the retraction of the drug from the market seems to be discouraging for clinical studies in POMS. However, in early 2012, Gorman and colleagues described seven patients with pediatric-onset MS with clinical and MRI disease activity, although they had been previously treated with first-line disease-modifying therapy. The POMS patients received IV Daclizumab, 1 mg/kg monthly. According to the authors, Daclizumab therapy, primarily in combination with interferon, achieved a reduction in ARR and contrast-enhancing lesions, as well as a reduction in, or stabilization of, Expanded Disability Status Scale (EDSS) scores in all patients. However, four patients experienced relapses and new contrast-enhancing lesions during daclizumab treatment. Of note, no significant adverse effects occurred [76].

## 6. Anti-CD52 Agents: Alemtuzumab

Alemtuzumab is a humanized monoclonal antibody targeting CD52, approved for the treatment of RRMS in adult-onset MS [77].

The clinical efficacy of Alemtuzumab in adult MS patients has been well demonstrated in phase III clinical studies, namely CARE-MS 1 (NCT00530348) and CARE-MS 2 (NCT00548405) [77,78], with the main safety and tolerability concerns being infusion-associated reactions, mild-to-moderate infections, and autoimmune adverse events (AEs), such as thyroid disorders and immune thrombocytopenia [79].

Regarding the use of Alemtuzumab in POMS, studies are limited. However, very recently, the results from the LemKids study (NCT03368664) were published. LemKids was a multicenter, multinational, single-arm, open-label, switch (from ongoing DMT to alemtuzumab treatment) study in POMS RRMS patients (aged between 10 and 18 years) with disease activity on DMT. The primary endpoint was a comparison of the number of new/enlarging T2 lesions on brain MRI between the prior DMT period and alemtuzumab treatment.

Of 46 screened patients, 16 were enrolled; 12 completed the prior DMT-treatment period, and 11 received Alemtuzumab, of whom 7 completed treatment. Patients on Alemtuzumab developed fewer new/enlarging T2 lesions compared with prior DMT (7 vs. 178, relative risk (95% confidence interval): 0.04 (0.01–0.14)). No significant pharmacodynamic changes or safety concerns were noted [80], in accordance with previously published data derived from small case series and case reports [81,82]. Additionally, these data were in line with previous studies exploring the safety and tolerability of Alemtuzumab in AOMS patients [77], as no deaths and new safety signals have been reported [83,84].

Unfortunately, the LemKids study was prematurely terminated after the European Medicines Agency Article-20 pharmacovigilance review of Alemtuzumab use in adult RRMS patients, which restricted its use due to safety concerns including serious cardiovascular reactions, newly identified autoimmune hepatitis, and hemophagocytic lymphohistiocytosis [85]. When the European Medicines Agency (EMA) safety review began in May 2019, LemKids recruitment was voluntarily stopped. Temporary limits on Alemtuzumab in adult patients were removed in January 2020 after the summary of product features was updated. However, LemKids recruitment was permanently closed because of the COVID-19 pandemic and continued difficulties with poor recruitment rates.

## 7. Future Perspectives for B-Cells Targeting Therapies in the Context of MS

**Figure 3:**
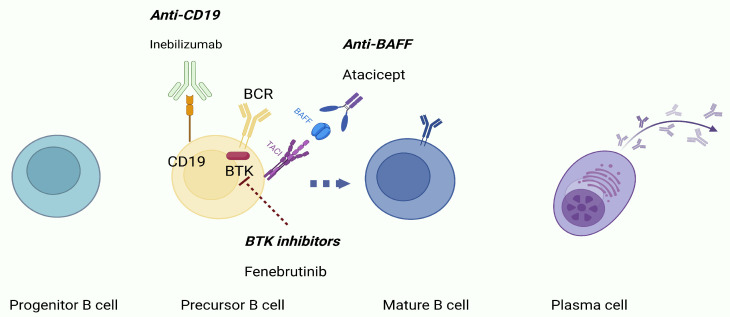
B-cell ontology and candidate B-cell-targeting therapies for adult-onset and pediatric-onset multiple sclerosis treatment.

Over the last decade, a growing body of evidence has revealed the major role of B-cells and related molecular pathways such as BAFF and Bruton’s tyrosine kinase (BTK) in MS pathophysiology, leading to exponentially increased research efforts for the development of B-cell-targeting therapies for MS treatment (Figure 3). The possible success of these therapeutic agents in clinical trials conducted on adult MS patients can lead to novel treatment strategies for the management of POMS also.

### 7.1. Targeting CD19

Anti-CD19 agents seem to represent a promising future therapeutic approach. CD19 belongs to the Ig superfamily, along with CD21, CD82, and CD225. CD19 contributes to the formation of a multimolecular signal transduction complex that ultimately leads to the activation of phosphoinositide-3 (PI-3) kinase [86,87]. Compared to CD20, CD19 is expressed on B-cells of earlier developmental stages, as well as in more antibody-secreting cells [88]. In addition to having a broader expression during B-cell stages of development and differentiation, CD19, unlike CD20, is selectively expressed on B-cells but not on T-cells [89].

A phase I study assessing the pharmacokinetic (intravenous and subcutaneous) profile of Inebilizumab, a humanized fucosylated IgG1κ anti-CD19 mAb [90], has been conducted in adult patients with relapsing MS showing encouraging results [91]. However, no phase III trials for AOMS are currently underway, while similar studies for POMS are absent.

### 7.2. Targeting BAFF Axis

The role of BAFF in systemic autoimmunity is well documented [92,93,94], and agents targeting the BAFF axis have been widely used for the treatment of autoimmune diseases including systemic lupus erythematosus (SLE), rheumatoid arthritis (RA), and Sjögren’s disease (SjD) [95]; however, only in the last years has the contribution of BAFF to MS development [32,33,96] and therapy been appreciated [97].

Atacicept is a human recombinant fusion protein consisting of a human IgG Fc portion and the extracellular domain of the (transmembrane activator and CAML interactor) TACI receptor that binds both BAFF and a proliferation-inducing ligand (APRIL) [98], blocking the binding of BAFF and APRIL to TACI, (both membrane-bound and in soluble form) as well as, to a lesser extent, the other receptors of the BAFF-APRIL system (BAFF-R and B-cell maturation antigen, BCMA) [99]. Unfortunately, when the efficacy and safety of atacicept were assessed in a phase II, double-blind, placebo-controlled trial, the study was prematurely terminated due to an increase in inflammatory disease activity, despite immunoglobulin and naïve B-cell decreases [100]. Up to today, no clinical trials in AOMS or POMS are in progress.

### 7.3. Bruton’s Tyrosine Kinase Inhibitors (BTKis)

**Figure 4:**
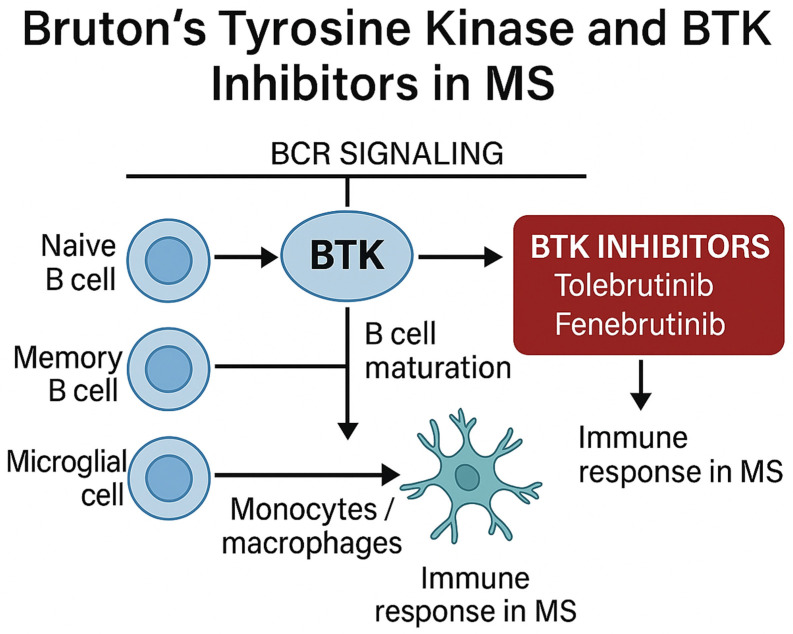
Schematic overview of the role of Bruton’s tyrosine kinase and its inhibitors in multiple sclerosis.

Bruton’s Tyrosine Kinase (BTK) is an integral part of the BCR signaling cascade that affects B-cell activation and is essential for B-cell maturation and their ultimate, terminal differentiation into memory or plasma cells; it is also necessary for the entry of B-cells into follicular structures [101]. BTK mediates signaling from several cell surface receptors, including BCR, FcγR, and TLR, to downstream molecules crucial to immune cell function. Since BTK is essential for coupling extracellular stimuli to the immune response in both adaptive immunity (B-cells) and myeloid phagocytic cells (CNS microglia and bone marrow-derived monocytes/macrophages), inhibition of BTK presents the opportunity to modulate adaptive and innate immune responses linked to MS pathophysiology [102]. Given the pivotal role of BTK in B-cell ontology as well as the increased expression of BTK by microglia, and that its expression increases in the brain after demyelination [103], BTK inhibitors hold promise for an effective MS therapy (Figure 4).

In this context, Evobrutinib was the first BTK inhibitor (BTKi) to be tested as a monotherapy in relapsing MS in a double-blind, placebo, and active control group (dimethyl fumarate) phase II trial [104]. Evobrutinib initially showed efficacy, as MS patients receiving a dose of 75 mg had significantly fewer gadolinium-enhanced lesions between 12 and 24 months of treatment compared to the placebo group [104]. Moreover, Evobrutinib was well tolerated, with mild adverse effects including nasopharyngitis, alanine aminotransferase, and aspartate aminotransferase level elevation. These results were confirmed in another 48-week, double-blind, phase II trial (NCT02975349) [105]. However, Evobrutinib failed to demonstrate superior efficacy compared to Teriflunomide in two phase III clinical trials for RRMS. Specifically, according to the published data, the ARRs were similar between the two drugs in the EVOLUTION clinical trials, suggesting that Evobrutinib did not provide a significant benefit in reducing relapses compared to existing treatments [105].

Additionally, treatment-emergent adverse events (TEAEs) were more frequent among subjects treated with Evobrutinib compared to those treated with Teriflunomide with high liver enzyme values and headache being the most common [105].

Likewise, three phase III studies investigating the efficacy and safety of another BTK inhibitor (BTKi), Tolebrutinib, on RRMS (NCT04410991, GEMINI2), primary progressive MS (PPMS) (NCT04458051, PERSEUS), and non-relapsing SPMS (NRSPMS) (NCT04411641, HERCULES) have recently concluded. According to the recently published data, non-relapsing secondary progressive MS patients treated with Tolebrutinib displayed significantly lower disability progression compared to their placebo counterparts [106]. However, Tolebrutinib failed to prove its superiority compared to Teriflunomide in the reduction of ARR among patients with RRMS [107]. Minor bleeding [107] and significantly higher (three times higher than normal) alanine transferase levels [106] were the most common adverse events.

Additionally, two ongoing trials are testing the BTKi Fenebrutinib on AOMS patients: a randomized, double-blind, placebo-controlled study exploring the efficacy of Fenebrutinib in adult RRMS patients (NCT05119569) and a phase III multicenter, randomized, double-blind, double-dummy, parallel group trial evaluating the efficacy and safety of Fenebrutinib compared to Teriflunomide in adult RRMS patients (Eudract number: 2020-001168-2).

BTK inhibitors may hold promise in the pediatric population due to their oral administration, which enhances treatment adherence, and their dual capacity to modulate both adaptive and innate immune responses. By targeting B-cells and CNS-resident microglia, BTK inhibition offers a unique therapeutic mechanism that may address the highly inflammatory environment characteristic of POMS [42,108]. Furthermore, their reversible mode of action and shorter systemic persistence may present a favorable safety profile in the developing immune systems of children, minimizing prolonged immunosuppression compared to anti-CD20 monoclonal antibodies [105]. Given that BTK expression is critical for B-cell maturation and signaling [108], as well as for microglial activation in demyelinated lesions and B-cell follicles in the meninges [42], these inhibitors provide a rational, targeted approach.

## 8. Conclusions

The evolving landscape of DMTs [76,109,110] for POMS highlights a shift toward early, high-efficacy treatment to mitigate irreversible CNS damage [55,69,76,111]. Among these therapies, B-cell-targeting therapies and especially the anti-CD20 agents have demonstrated significant efficacy in adult MS and are emerging as promising options for POMS [69]. However, their use in the pediatric population remains largely off-label, necessitating further research to establish their long-term safety and effectiveness [112,113].

Tailoring optimal treatment strategies for POMS with B-cell-targeting therapies requires an in-depth understanding of the disease’s unique immunopathogenesis and the specific role of B-cells in its progression. While preliminary data suggest that anti-CD20 therapies may offer substantial clinical benefits, additional controlled studies are needed to refine treatment protocols, assess long-term outcomes, and ensure safety in the pediatric MS population.

Future research should focus on optimizing treatment initiation, monitoring strategies, and potential combination therapies to improve disease control and long-term neurological outcomes in children with POMS, bridging the time gap of these therapies’ initiation in this population compared to the AOMS population worldwide.

Apart from immunotherapies that have already been tested and administered in adult MS populations, a major, advanced future direction could be the development of other novel agents tailored for POMS with high efficacy, fewer adverse events, and precision therapeutics in the evolving artificial intelligence era.

## Figures and Tables

**Table 1:** Key differences between POMS and AOMS.

Clinical Feature	POMS	AOMS	References
Age at Disease Onset	<18 years	≥18 years	[21,22,23,24]
Initial Disease Course	Almost exclusively RRMS	Relapsing or progressive	[21,22,23,24]
Inflammatory Activity	Higher—more aggressive inflammatory onset	Often less inflammatory at the onset	[21,22,23,24]
Relapse Rate	Higher (2.3–2.8 times more frequent)	Lower	[21,22,23,24]
Number of Demyelinating Lesions	Increased	Fewer	[21,22,23,24]
Axonal/Neuroaxonal Damage	More pronounced	Less pronounced	[24]
Disability Accumulation Rate	Slower initially	Faster initial progression	[21,22,23,24]
Transition to SPMS	Approximately 10 years earlier than in AOMS	Later transition	[22]
Final Disability Milestone	Reached at a younger age due to early onset	Reached at older age	[21,22,23,24]
HLA-DRB1*	Predominantly HLA-DRB1*03 and classically HLA-DRB1*15:0 *in some populations*	HLA-DRB1*15:01	[14]

RRMS: relapsing–remitting multiple sclerosis; SPMS: secondary progressive multiple sclerosis; POMS: pediatric-onset multiple sclerosis; AOMS: adult-onset multiple sclerosis; HLA: human leucocyte antigen.

**Table 2:** Summary of B-cell-targeting therapies in multiple sclerosis.

Therapy	Mechanism of Action	Efficacy in POMS	Safety Concerns	Current Clinical Trials Status in POMS
Rituximab	A chimeric anti-CD20 monoclonal antibody that depletes B-cells through ADCC, CDC, and apoptosis	Shown to reduce relapse rates, MRI activity, and disability progression in retrospective studies	Infusion reactions, infections, rare risk of PML	No approved trials in the pediatric MS population
Ocrelizumab	Humanized anti-CD20 monoclonal antibody with enhanced ADCC activity	Promising preliminary results from the OPERETTA trials; sustained B-cell depletion and reduced relapses	Infusion-related reactions, infections, rare risk of malignancies	Active, not recruiting
Ofatumumab	Fully human anti-CD20 monoclonal antibody with higher CDC activity than rituximab	Shown to reduce ARR and MRI lesions in adults; limited POMS data	Injection-site reactions, infections, possible immune suppression	Ongoing phase II trial in POMS (NCT04926818, NEOS)
Alemtuzumab	Anti-CD52 monoclonal antibody leading to depletion of T- and B-cells	Small studies suggest effectiveness, but safety concerns limit the use	Autoimmune disorders (thyroid, ITP), infusion reactions	Early termination of the NCT03368664 LemKIds study
BTK Inhibitors	BTK, reducing B-cell activation and microglia-mediated inflammation	Early trials show promise in adult MS; no POMS-specific trials yet	Mild liver enzyme elevations, infections	No approved pediatric trials yet
Anti-CD19 therapies (Inebilizumab)	Targets CD19, affecting a broader range of B-cell subsets than anti-CD20 therapies	Early-phase adult MS trials are ongoing; no pediatric data yet	Immunosuppression, infection risk	No approved pediatric trials yet
Anti-CD25 therapies (Daclizumab)	Monoclonal antibody targeting interleukin (IL)-2 receptor alpha (IL-2Rα) chain (CD25), leading to a reduction in activated T-cells	No clinical trials, only case series	Fatal encephalitis in adult clinical trials	No approved pediatric trials

POMS: pediatric-onset multiple sclerosis; PML: progressive multifocal leukoencephalopathy; ADCC: antibody-dependent cellular cytotoxicity; MRI: magnetic resonance image; BTK: Bruton’s tyrosine kinase.

**Table 3:** A brief overview of clinical trials regarding B-cell-targeting agents on pediatric-onset multiple sclerosis patients.

Clinical Trial	Therapy	Study Design	Primary Outcomes	Status
OPERETTA I (NCT04075266)	Ocrelizumab	Phase II open-label	Pharmacokinetics, safety, ARR reduction	Active, not recruiting
OPERETTA II (NCT05123703)	Ocrelizumab vs. Fingolimod	Phase III randomized	ARR reduction, MRI outcomes	Active, not recruiting
NEOS (NCT04926818)	Ofatumumab vs. Siponimod vs. Fingolimod	Phase II randomized	ARR reduction, MRI lesion burden	Active, not recruiting
LemKids (NCT03368664)	Alemtuzumab	Open-label	Reduction in MRI activity, ARR reduction	Early termination

ARR: annual relapse rate; MRI: magnetic resonance image.
